# Human Performance in Competitive and Collaborative Human–Machine Teams

**DOI:** 10.1111/tops.12683

**Published:** 2023-07-13

**Authors:** Murray S. Bennett, Laiton Hedley, Jonathon Love, Joseph W. Houpt, Scott D. Brown, Ami Eidels

**Affiliations:** ^1^ School of Psychology The University of Newcastle; ^2^ Department of Psychology University of Texas at San Antonio

**Keywords:** Human–AI teaming, Workload capacity, Group performance, Collaboration, Competition

## Abstract

In the modern world, many important tasks have become too complex for a single unaided individual to manage. Teams conduct some safety‐critical tasks to improve task performance and minimize the risk of error. These teams have traditionally consisted of human operators, yet, nowadays, artificial intelligence and machine systems are incorporated into team environments to improve performance and capacity. We used a computerized task modeled after a classic arcade game to investigate the performance of human–machine and human–human teams. We manipulated the group conditions between team members; sometimes, they were instructed to collaborate, compete, or work separately. We evaluated players' performance in the main task (gameplay) and, in post hoc analyses, participant behavioral patterns to inform group strategies. We compared game performance between team types (human–human vs. human–machine) and group conditions (competitive, collaborative, independent). Adapting workload capacity analysis to human–machine teams, we found performance under both team types and all group conditions suffered a performance efficiency cost. However, we observed a reduced cost in collaborative over competitive teams within human–human pairings, but this effect was diminished when playing with a machine partner. The implications of workload capacity analysis as a powerful tool for human–machine team performance measurement are discussed.

## Abstract 2

We used workload capacity analysis to quantitatively evaluate group performance of human–machine dyads. Groups completed a dynamic team‐based task under a collaborative and competitive team. We found that the group processes required for collaboration and competition resulted in costs to group performance, but these costs were reduced in human teams compared to human–machine teams.

## 1 Human performance in competitive and collaborative human–machine teams

Many modern tasks require groups of individuals or human–machine (HM) groups to complete. Groups outperform individuals on complex tasks by having greater available resources and effective intragroup interactions. Successful group performance occurs when the group develops *collective intelligence* (Woolley, Chabris, Pentland, Hashmi, & Malone, 2010). Collective intelligence emerges when group members share an understanding of the task, each member's abilities, and the tasks being completed by each member (Gupta & Woolley, 2021). It follows that group members must interact and share information between group members to develop such an understanding. While interactions between group members can aid task completion, inefficient interactions can also lead to costs to the group's potential performance (Hsieh et al., 2020; Steiner, 1966). The rise of machine agents to aid task performance provides powerful opportunities for performance gains, but the efficiency of HM interactions remains ambiguous.

Maximizing the performance of human and HM teams is critical but requires the accurate evaluation of group performance. Accurate and quantitative measurement of group performance is difficult. The trade‐off between the performance gains associated with additional group resources and efficient group interactions with the costs derived from inefficient interactions limits accurate evaluation of a group's overall performance. For example, two individuals may outperform an individual on a task as the dyad has access to double the resources. However, inefficient group processes may inhibit the group from producing the group's potential performance even when the group output is greater than an individual. The difference between a team's potential output and actual output is a concept known as *process loss* (Steiner, 1966), to which we refer as performance *cost*.

The current study aims to quantitatively evaluate HM group performance and characterize the efficiency of the group interactions underlying performance. We implement *workload capacity analysis* to evaluate group performance, a modeling tool typically utilized in cognitive science to assess information exchange between internal cognitive processes (Algom et al., 2015; Townsend & Nozawa, 1995). To evaluate the efficiency of group interactions, we subject HM dyads to a task requiring different group interaction processes. Specifically, we instructed dyads to collaborate, compete, or work separately. We then compare HM group performance to human–human teams (HH) on the same task. The following section elaborates on workload capacity analysis as an effective measurement tool for HM teams and how researchers can use this measure to evaluate the effects of a group's internal processes on performance. We then introduce the group processes observed in collaborative and competitive human groups and evaluate how these different processes could affect HM group performance.

### 1.1 Workload capacity analysis

Accurate and quantitative measurement of team performance, particularly in HM teams, is critical to understanding and evaluating machine and team design characteristics and the processes used within these teams (National Academies of Sciences, Engineering, & Medicine, 2022). Although tasks completed by teams often lead to increased overall output, performance gains can be deceptive—increased team performance relative to an individual is possible with and without interactions between group members. The statistical facilitation of group performance occurs when task performance improves without any interaction between group members (e.g., Surowiecki, 2005). Groups can also perform satisfactorily with team members performing below their best (Zajonc, 1965) or when interactions between group members inhibit an individual's ability to complete task requirements (Steiner, 1966). Critically, costs to team performance via reduced individual performance or process loss can be masked when the group's performance exceeds an individual's or the task is completed satisfactorily.

The statistical facilitation of performance observed in group tasks is a troublesome challenge, making the accurate evaluation of group performance and the efficiency of the underlying group processes difficult. However, *workload capacity analysis* contains the appropriate machinery to overcome these obstacles, providing a quantitative and objective assessment of HM team performance. We detail the modeling approach below in the methodology but present a conceptual overview here.

Workload capacity analysis generates a quantifiable metric of group performance by scaling the group performance against the group members' combined performances. A group's performance efficiency is then identified by comparing this scaled group metric against a theoretical benchmark—the performance expected of a group if the group's constituent members completed the task without interaction. Workload capacity analysis is typically used in cognitive science to identify and characterize information exchange between internal cognitive processes (Algom et al., 2015; Townsend & Nozawa, 1995). Workload capacity analysis has recently helped demonstrate the utility of HM collaborations by assessing an *individual's* response behaviors to automation aids under different automation levels (Yamani & McCarley, 2016), aid display formats (Scott‐Sharoni et al., 2021), and task difficulty (Yamani & McCarley, 2018; Zinn, Yamani, Houpt, & Scott‐Sharoni, 2018).

However, and of most interest to the current work, workload capacity analysis can also be extended beyond the cognitive domain to evaluate group performance by quantifying and characterizing the efficiency of group interactions *between* individuals (Hsieh et al., 2020; Yamani & McCarley, 2016). To do so, we conceptualize individuals as distinct information processors, and the team performance represents the overall system output. Framing individual group members and group performance in this way allows researchers to access the powerful analytical machinery offered by workload capacity. The approach presented here opens a promising avenue for the empirical measurement and assessment of performance and the efficiency of information exchange within HM teams.

For example, collective intelligence is a recognized construct in HM teams and can be summarized by a shared awareness of team‐member skills and the tasks under completion. Collective intelligence has typically been examined within human teams and has been shown to relate positively to group performance on various tasks (Gupta & Woolley, 2021). The collective intelligence of HM teams, or Collective Human Machine Intelligence (COHUMAIN; see other papers in this special issue), is a newly developing field as intelligent machines evolve. However, advancing artificial intelligence systems' complexity or task capacity does not necessarily improve overall team performance (Bansal et al., 2019) as they cannot *efficiently* share, produce, and integrate group‐level knowledge. By definition, the emergence of a group's collective intelligence requires interactions and the exchange of group‐relevant information between individuals. It is, therefore, reasonable to expect group performance by HM teams to be poorer than HH teams on the same task; however, this may not always be the case. As intelligent machines become prevalent in group tasks, particularly in safety‐critical tasks, it is essential to evaluate HM team performance effectively. Workload capacity analysis can be used in such contexts to evaluate specific interactions between group members and the extent to which the emergence of collective intelligence affects group performance.

### 1.2 Collaborative and competitive groups

Consider a natural disaster event where two groups of individuals are working on a search and rescue task for missing individuals. The first group, a collaborating pair, operates by dividing responsibilities within the group. One person may operate a helicopter while the other scouts the environment for missing persons, facilitating rapid and accurate coverage of the disaster zone. Performance costs or process losses may emerge if, for example, team members fail to coordinate actions effectively or communication needs to be clearer between members. The second group operates in a competitive context, where rewards per found person incentivize individual team members. One person may still fly the helicopter, but the same individual might also engage in additional group tasks, such as scouting. The competitive group might then observe performance benefits via redundancy gains where, for example, a target that has been missed by group member A is located when group member B intentionally surveys their competitor's search space. However, groups can experience performance costs when group members forego their primary tasks to explore their competitor's search space.

Performance should theoretically improve if group members develop collective intelligence in all cases. That is, performance on this task will improve when the pilot and scout share a common goal, understand the skills of each member, and know the tasks being completed by each group member (Gupta & Woolley, 2021). Both collaborative and competitive groups can develop such an understanding despite different group interaction processes. However, how the differences in the unique group processes of collaboration and competition equate to differences in group performance is still being determined in HM teams.

Consider a scenario where either team comprises a pilot and a machine agent scout designed to scan the environment. Does including a machine agent teammate inhibit the group's potential performance, and do the group conditions mediate these potential benefits and costs? Most group performance research is conducted under collaborative group conditions, so the effects of collaborative conditions can already be estimated. In spatial tasks, for example, HH collaborative groups aim to minimize individual workload, thereby maximizing group performance, by spreading task load between members (Brennan, Chen, Dickinson, Neider, & Zelinsky, 2008; Yamani et al., 2017). Collaborative teams also provide cues to team members that aid performance by prompting teammates toward solutions (Brennan & Enns, 2015b). HH teams demonstrate coordination and anticipation of teammate behavior (Demir, McNeese, & Cooke, 2017; Grimm, Demir, Gorman, & Cooke, 2018; McNeese, Demir, Chiou, & Cooke, 2021), two key behaviors observed in teams with collective intelligence (Gupta & Woolley, 2021). Social factors are also relevant to HM research. For instance, Walliser, de Visser, Wiese, and Shaw (2019) found that social interaction via team building exercises with a machine agent improved strategy adoption and performance in a warship defence task. Zhao et al. (2022) found task improvements when machine agent aids adapted to the ability level of the human agent.

In contrast, group performance might also improve under competitive conditions. Where collaborating individuals are inclined to divide their search space (e.g., Brennan & Enns, 2015b, 2015a), competing individuals monitor fellow group members to maximize individual performance (Bennett et al., 2020; Niehorster et al., 2019). Competitive group environments can result in enhanced motivation (Camerer & Hogarth, 1999), states of heightened arousal (Adam et al., 2017), and increased between‐member monitoring—leading to increased accuracy via redundancy gains (Niehorster et al., 2019). However, competition can also lead to erratic behavior (Niehorster et al., 2019) as individuals attempt to chance on solutions. Competitive group environments also require individuals to evaluate and incorporate competitor behaviors to formulate their best actions. The increased monitoring between group members can lead to suboptimal decision‐making as relevant task information is missed (Bennett et al., 2020).

The current research on HM teaming indicates promising collaborations between human and intelligent machine agents. However, the effects of different social contexts on performance in HM teams are unknown. Collaboration and competition between members offer potential performance gains and costs in human teams, but how the benefits and costs of competitive groups translate to HM teaming conditions is unclear. Borrowing from human‐team research, members of a competitive HM team might aim to maximize individual performance, but at the cost of spending additional resources monitoring group members (Bennett et al., 2020; Niehorster et al., 2019). Given the importance of cohesion in HM teams, Correia, Melo, and Paiva (2022), a competitive human–machine team that operates to maximize individual performance may result in costs to the group's performance potential. However, given the powerful capabilities of intelligent machines, intelligent machine agents in a competitive environment may perform to their capacity, unhindered by limitations required to support human teammate abilities (cf. Zhao et al., 2022). Additionally, a competitive environment may help improve human performance through an increased motivation to succeed against the machine agent (Adam et al., 2017; Camerer & Hogarth, 1999).

Overall, the literature indicates that collaborative groups working cohesively will produce reliable performance improvements, whereas competitive group conditions can benefit group performance gains via increased individual motivation but risk redundant actions when trying to outperform their competitor. Accurate and quantitative performance evaluation in these group conditions allows researchers and machine agent design engineers to evaluate the optimal conditions for HM groups. The following section describes a study where we evaluated the group performance of HM teams under collaborative and competitive group conditions.

### 1.3 The current research

Groups can produce greater performance than individuals, and groups that interact to develop collective intelligence are associated with increased performance. However, ineffective intragroup interactions also lead to performance costs. Interactions between group members will be distinct depending on the group's social conditions (collaborative or competitive) and the group composition (HH or HM). Differences in the intragroup processes will affect the shared understanding of the group and the task held by group members. Such differences may result in costs to the group's performance. However, performance costs can be masked by the group's overall performance, making it difficult to quantitatively and objectively evaluate different group compositions and conditions. We implement workload capacity analysis as a measurement tool to overcome these challenges of group performance assessment. The primary research aim for this study is to objectively and quantitatively examine human performance in HM teams in collaborative and competitive group conditions compared to HH teams.

To address this aim, we developed a novel online platform, *Team Spirit*, to empirically examine group performance and the intrateam interactions used within different group social conditions. *Team Spirit* is a simple arcade‐like game where two players, either humans or a human and a machine bot, complete a primary task of deflecting falling balls with their paddles while also completing a secondary signal detection task, the detection response task. HH and HM teams were instructed to complete the task under different group conditions: to work separately, collaboratively, or competitively. We further manipulated the task workload across trials by varying the number of balls presented per trial. The novel platform allows precise measurement of individual and group performance and moment‐by‐moment monitoring of player positions, providing a proxy for their behavioral strategies. We present three main‐effect hypotheses corresponding to our experimental team type, group conditions, and workload manipulations. First, the primary task performance of HM dyads will be less than that of HH dyads. Second, for human and HM teams, primary task performance will be greatest in the collaborative condition, followed by the competitive, then separate conditions. Finally, we expect performance to decrease as the task workload increases for all team types and group conditions.

## 2 Methods

### 2.1 Participants

Participants were recruited via the online platform Prolific and were compensated £5. One‐hundred and thirty‐eight participants completed the task in HH dyads and 296 other participants completed the HM task. Following data cleaning (see the Measures section), the final HH participant pool was 126, or 63 dyads (mean age = 34.8, *SD* = 11.4), and 288 HM teams (mean age = 41.8, *SD* = 13.0). All participants reported having a normal or corrected‐to‐normal vision and were fluent in English. The Human Research and Ethics Committee at the University of Newcastle, Australia, approved this research.

### 2.2 Design

Team type (HH, HM) was manipulated between dyads. Group conditions (separate, collaborative, competitive) and workload (low, medium, high, very high) were manipulated within subjects. We recorded the miss rate and player coordinates in the primary task (described below) and the accuracy and response times to the detection response task.

Participants in the HM teams were paired with and counterbalanced across one of three machine agents. We briefly describe the machine agents used in this task in the following subsections but, following null effects between agent types, do not include agent types as factors in the current analysis. Similarly, machine agent transparency was included in the design as a counterbalanced between‐subjects condition but was also found not to affect performance and is similarly aggregated. Further details, analyses, and results are available at https://osf.io/mtazf/.

The task was completed over three blocks, where each block instructed participants to play strategically according to counterbalanced group conditions (e.g., collaborate). Machine agent types were counterbalanced across participants in the HM task such that 96 participants were allocated across the three machine agent condition combinations.

### 2.3 Task

*Team Spirit* is a novel platform developed to examine group performance in a dynamic task. The task is presented as a simple arcade‐like multiplayer game where two players must deflect falling balls with their paddles while completing a secondary task, the detection response task (DRT). We include further details of the DRT in the Supporting Information but do not present a complete analysis of this data in the current paper. Participants were presented with the same game content simultaneously; however, there were no direct forms of communication between participants.

The task was programmed using Python 3.8 for the server‐side content and JavaScript for client‐side presentation and participant interaction. Participants progressed through instruction screens and indicated their readiness to begin trials by pressing the space bar. Right‐handed participants moved the paddle with the left and right arrow keys and made DRT responses with the *z* key. Left‐handed participants used the *z* and *x* keys for left and right movements and made DRT responses with the up arrow. Paddles only moved when a key was pressed and held.

#### 2.3.1 Team spirit game

**Fig. 1:**
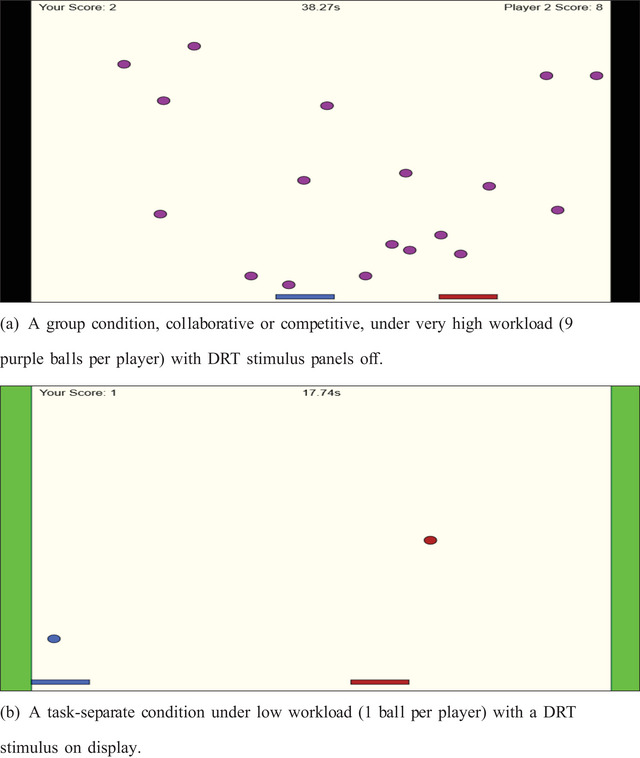
Example playing screens under different group and workload conditions and the DRT stimulus.

Participants in HH teams were assigned to control the left or right paddles according to their log‐in order, while participants in the HM team were randomly allocated starting positions. A player's paddle was always presented in red and their teammate in blue. Paddle movement across the screen was only limited by the vertical edges of the DRT panels, such that the position of one player did not inhibit the movement of the other. Balls were colored purple in the collaborative and competitive conditions (Fig. 1a) but were separated into red (player) and blue (teammate) in the separate condition (Fig. 1b).

Players were awarded a *hit* when balls rebounded off a paddle and a *miss* when a ball passed the bottom of the screen. Each hit rewarded a player with +1 to their individual score in the separate and competitive conditions or +1 to the combined team score in the collaborative condition. Hits and associated scores were split equally if players hit the same ball simultaneously in collaborative or competitive conditions.

#### 2.3.2 Playing space

All displayed content was presented to participants in an 800×600 pixel (px) frame adjusted to the relative width and height of the participant's screen. DRT signals were displayed to participants via 40‐px‐wide panels on both vertical sides of the screen that spanned the height of the display. The playable space occupied the remaining space between the DRT panels (see Fig. 1). Note that player movements were unimpeded by the position of their teammate; a player's paddle would pass over their teammate when occupying the same playing space. To reinforce group conditions within trials, we presented the cumulative scores for both players separately in the separate and competitive conditions, and the combined team score was displayed in the collaborative condition.

### 2.4 Machine agent design

Participants in the HM task were paired with one of three agent types. The first agent was a reinforcement learning agent trained using hits as a reward (Watkins & Dayan, 1992). The second and third machine agents were designed with ideal‐observer constructs (Eidels & Gold, 2014; Geisler, 2003). Specifically, the second agent utilized ball intercept points and times until interception within the task and the third used the same algorithm but was coded to avoid the human player's position in the collaborative condition. Note that agents were not designed to be optimal task performers but rather to elicit human‐like performance outcomes based on the miss rates observed in the HH team data collection stage—an average of 0.05 and 0.65 in the low and very high workload conditions, respectively. As all bots were designed to produce similar performance outcomes, we aggregate data across the three agent types for the analysis in this paper. Extended details of these agent designs and performance analysis between the agent types are included in the Appendix and online at https://osf.io/mtazf/.

### 2.5 Measures

Data from the practice trials were not recorded. Exclusion criteria were set using performance in the low workload separate condition as a participant had only a single ball and no team responsibilities. HH and HM dyads were removed from analysis if any human participant in the team scored less than 50% accuracy to the DRT or above 50% miss rate in this condition. We implement both frequentist and Bayesian analysis.

#### 2.5.1 Primary task performance

Performance in the primary task was measured via miss rate, calculated as the complement of the hit rate (i.e., $1-\frac{hits}{hits+misses}$), where we used paddle‐ball collisions as hits and balls passing the bottom of the screen as miss events.

**Fig. 2:**
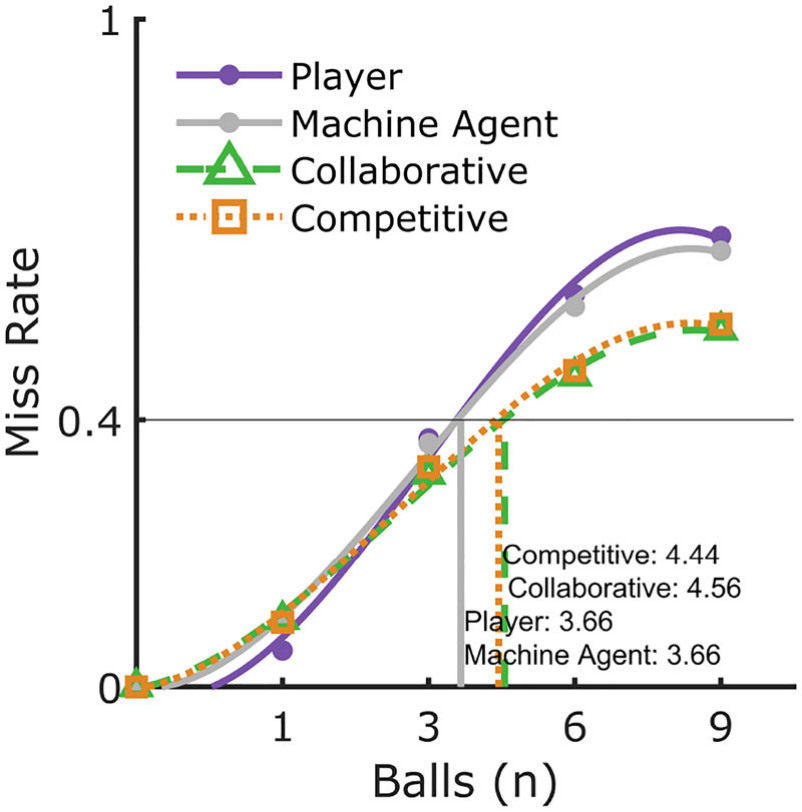
The number of balls maintained at a 40% miss rate for all players and teams was determined by fitting a line to miss rate data and determining the *x*‐value of the intersection between the fitted line and a 40% miss‐rate benchmark.

Along with miss rates, task performance was further analyzed via the number of balls maintained at any given moment—a simple transformation of miss rate data designed to account for changes in miss rate caused by the workload manipulation. Specifically, the *number of balls maintained* is calculated for each group type via a cubic polynomial interpolation to estimate the workload (number of balls) associated with a 40% miss rate (for explication, see Fig. 2). Such transformations are common in psychophysics experiments (Wichmann & Hill, 2001) and are conceptually similar to mean centering. This approach has two primary benefits. First, team and group types can be compared freely without extraneous effects of workload. Second, this transformation provides an intuitive way for assessing team efficiency by estimating the number of balls that could be maintained at some fixed accuracy level (here, 40%) across conditions. For example, if Player 1 maintains, on average, 4.3 balls at 40% accuracy and Player 2 retains 3.2 balls, the predicted team performance assuming no loss of efficiency, is their summed performance, 7.5 balls. Anything less implies the players are not as effective within a team as they are in isolation (Eidels, Townsend, Hughes, & Perry, 2015; Townsend & Eidels, 2011)

#### 2.5.2 Workload capacity coefficient

The workload capacity coefficient (C(p) ) is a modeling tool adopted from Systems Factorial Technology used here to characterize team processing efficiency as either *limited*, *unlimited*, or *super capacity* (Algom et al., 2015; Townsend & Nozawa, 1995). Here, we evaluate the performance efficiency of teams (HH and HM teams) by comparing the capacity coefficient to its embedded unlimited capacity independent parallel (UCIP) model. The UCIP model is a theoretical benchmark that represents a team's expected performance if all team members were working simultaneously and independently (Algom et al., 2015).

The benchmark is determined for a dyad at each workload level by taking the product of the team member's performance when completing the task separately. The efficiency of a team under different workloads or group conditions can then be evaluated via the capacity coefficient (C(p) ) by taking the difference between the group performance and the UCIP benchmark:

C(p)=p(A)×p(B)−p(AB).

For each dyad at each workload level, p(A) and p(B) represent the *miss rates* of team members 1 and 2 when completing the separate condition and p(AB) is the miss rate of a dyad in either the collaborative or competitive team environment (see Heathcote et al., 2015). Three outcomes can be derived from comparison to the UCIP model: scores above 0 represent supercapacity and indicate a collective benefit of the team, whereas scores below 0 represent limited capacity, indicating a cost of teamwork. Finally, scores equal to 0 represent unlimited capacity and indicate neither a cost nor gain of combining effort as a team.

#### 2.5.3 Measures of behavioral patterns

In addition to performance measures, we also assessed players' behavioral patterns to inform potential sources of team‐efficiency loss or gain. Paddle positions were recorded throughout the experiment and were used to calculate momentary distances and the proportion of highly correlated segments (HCS). *Momentary distance* was calculated as the mean absolute distance between players at each screen update in the game, which indicates spatial management strategies. For example, a dyad with high momentary distance operates in separate spaces more frequently, whereas low momentary distances suggest players utilize similar spaces. *HCS* is calculated as the proportion of trial time where cross‐correlated player movements were above a threshold of 0.97 (see Marcelino et al., 2020, for details) and is used to indicate coordinated actions within the dyad. For a comprehensive assessment of these measurements and other behavioral properties examined in this task, see Hedley et al. (2023).

### 2.6 Procedure

Participants accessed the experiment via Prolific. Initial instructions for all HH teams and the ambiguously informed HM teams informed participants that the game would be completed with another player. Participants in the HM teams in the machine‐informed condition were explicitly informed that their playing partner was a machine algorithm. The aim of the game (i.e., maximize ball deflections) was then described to participants. Participants then indicated their handedness to determine paddle control keys before playing a brief 25‐s low‐workload trial without the DRT.

The DRT was then introduced and participants were shown the relevant response key. Participants completed a 25‐s practice trial where *only* DRT stimuli were shown. This practice trial was followed by a full‐length medium workload practice trial (i.e., three balls for 45‐s). Participants completed all practice trials separately.

Participants were then provided instructions on the group condition of the upcoming block. The collaborative condition was established by instructing participants to “work together to score as high as you can” (toward a single, common score). In contrast, the competitive condition instructed participants to “try to outscore your opponent.” The “separate” condition required participants to play with separate targets and was constructed by informing participants that they could only interact with balls that matched their paddle color. This was also controlled via game mechanics whereby balls of the color of the opposing paddle would not deflect, simply passing through the paddle.

Blocks contained twelve 45‐s trials where each of the four workload conditions was presented in random order three times for a combined total of 36 trials across three blocks. Trials were separated by a minimum 5‐s break where each player's trial score and cumulative block score were presented. Following the forced break, the next trial began once *both* participants in a HH team had indicated readiness. Interblock breaks presented instructions for the next block within a forced 20‐s break. The experiment ran for approximately 40 min.

## 3 Results

### 3.1 Primary task team performance

#### 3.1.1 Miss rates

**Fig. 3:**
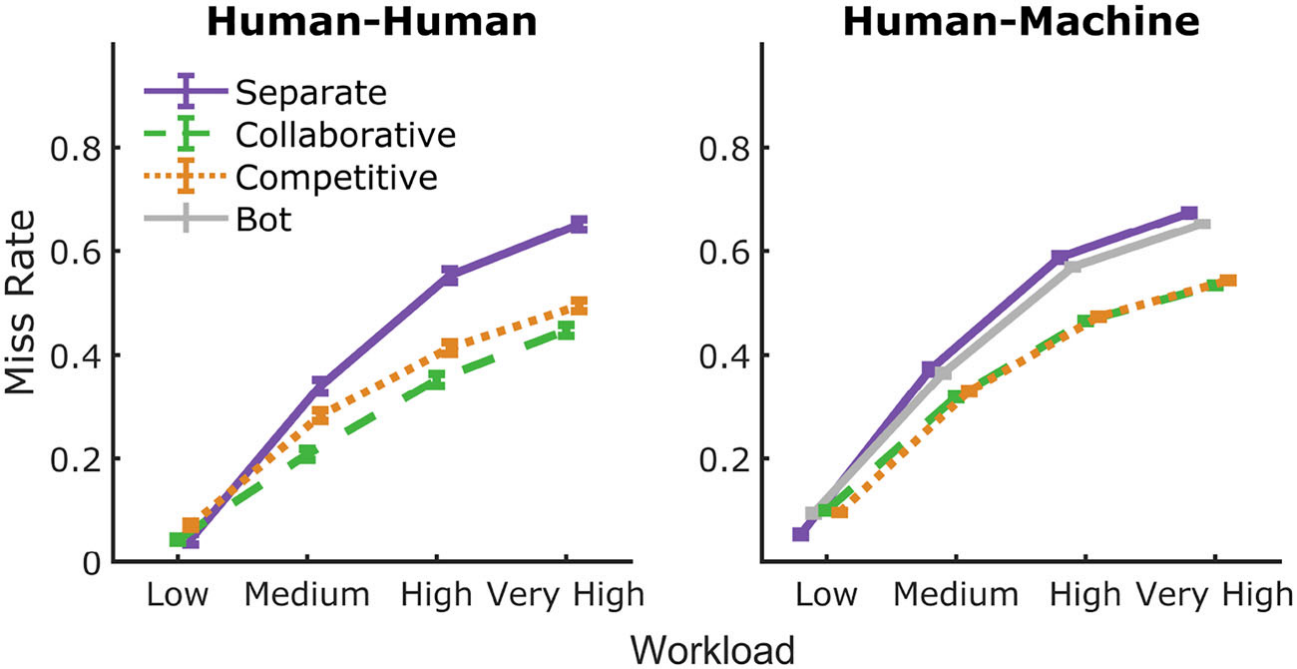
Primary task miss rates across group types and workload levels in HH teams (left) and HM teams (right). Lower miss rates indicate better performance. Miss rates increased with workload but are lowest overall in the collaborative team condition, followed by competitive teams and individual performances. Performance was similar across HH and HM teams in all conditions, but there was no difference between collaborative and competitive HM teams.

Fig. 3 shows mean miss rates across all conditions. Note that *lower* miss rates represent better performance. Machine agent miss rates in the separate condition were excluded, and player one and two separate condition miss rates were averaged within the HH dyads for analysis in a 2×3×4 mixed analysis of variance (anova). We include team composition (HH, HM) as the between‐subject factor and group type (separate, collaborative, competitive) and workload (low, medium, high, very high) as within‐subject factors.

All main effects and two‐way interactions were significant (team type: $F_{\left(1,349\right)}=37.41$, p<.001, $BF_{inclusion}>1,000$; group condition: $F_{\left(1.49,518.36\right)}=358$, p<.001, $BF_{inclusion}>1,000$; workload: $F_{\left(2.2,768.73\right)}=9,229$, p<.001, $BF_{inclusion}>1,000$). Miss rates were significantly lower (i.e., better performance) in HH teams when compared to HM teams (mean difference = −0.052, t=−6.12, p<.001, posterior odds >1,000).

Across both HH and HM teams, performance was greatest in the low workload condition and increased significantly across each increment of workload up to the very high workload level (all *p* < .001, posterior odds > 1,000). The significant interaction between workload and team types ($F_{\left(1.19,518.36\right)}=41.32$, p<.001, $BF_{inclusion}>1,000$) indicated that miss rates between HH and HM teams were not different in the low workload condition (*p* = .090) but were significantly higher in the HM teams for all other workload levels (all *p* < .001).

Miss rates were highest in the separate group condition but decreased significantly in competitive teams (t=−18.58, p<.001, posterior odds >1,000), and then again from competitive to collaborative teams (t=−7.42, p<.001, posterior odds >1,000). Of most interest to the study, where collaborative groups showed a clear performance improvement over competitive groups in the HH condition (mean difference = −0.053, *t* = −7.313, *p* < .001), we observed no significant difference between collaborative and competitive HM pairings (mean difference = −0.006, *t* = −1.87, *p* = .071). However, Bayesian analysis indicated that there was moderate evidence that miss rates had differed between the two team types (posterior odds = 14.77).

The contrary outcomes between analyses were examined further by collapsing data across workload and examining the interaction between team type and group condition (see Fig. 6, left). It should be noted that workload capacity analysis, reported below, also provides informative insight to supplement this finding. Results from a mixed anova showed a significant interaction between team type and group condition ($F_{\left(1,349\right)}=82.5$, p<.001, $BF_{inclusion}>1,000$), indicating that the performance benefit of collaboration over competitive teams was larger for HH teams than HM teams.

**Fig. 4:**
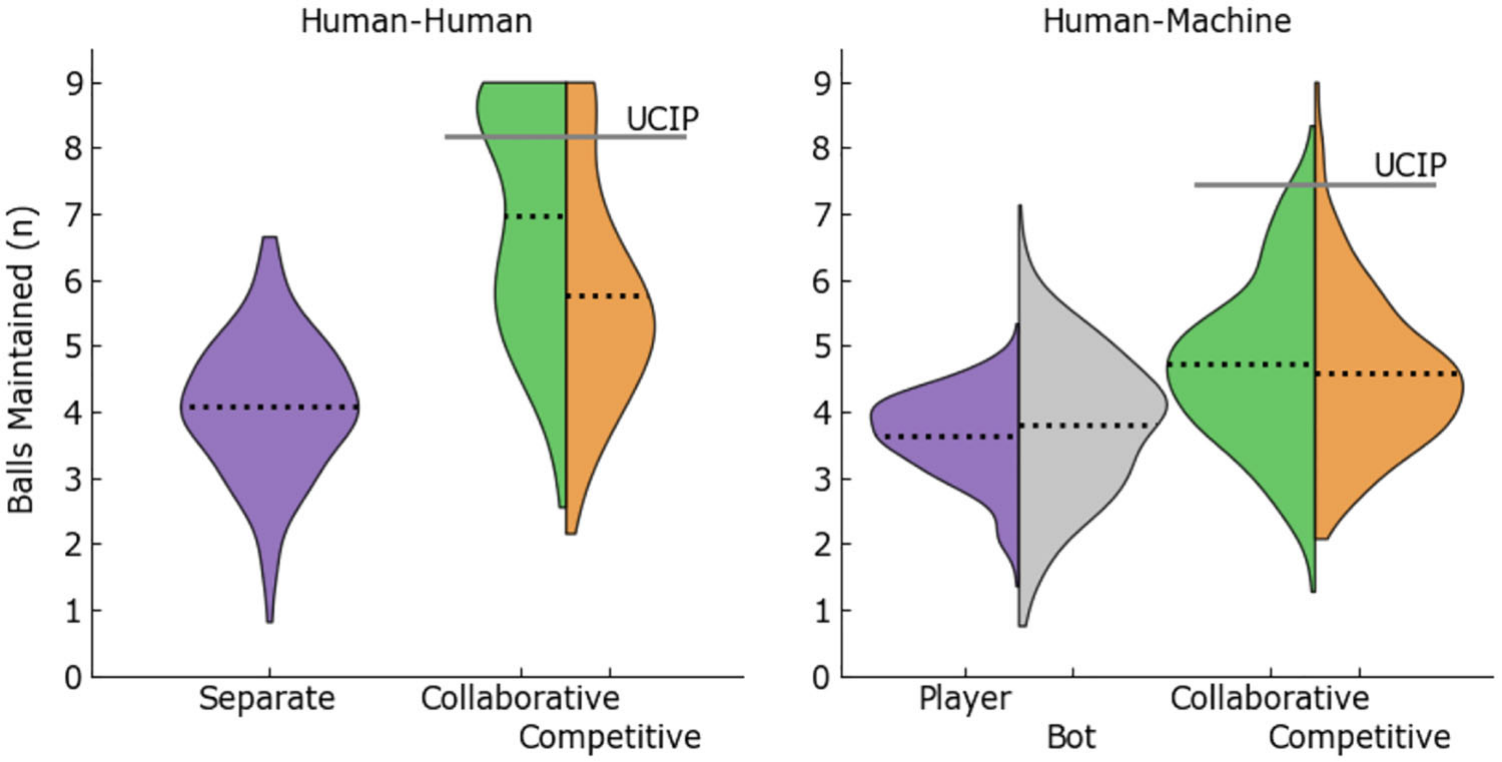
Distributions and means (dotted) of the number of balls maintained with a 40% miss rate for HH teams (left) and HM teams (right). Collaborative and competitive teams maintained more balls than individuals and bots but were again below the UCIP benchmark, indicating a cost of teamwork on team processing efficiency.

**Fig. 5:**
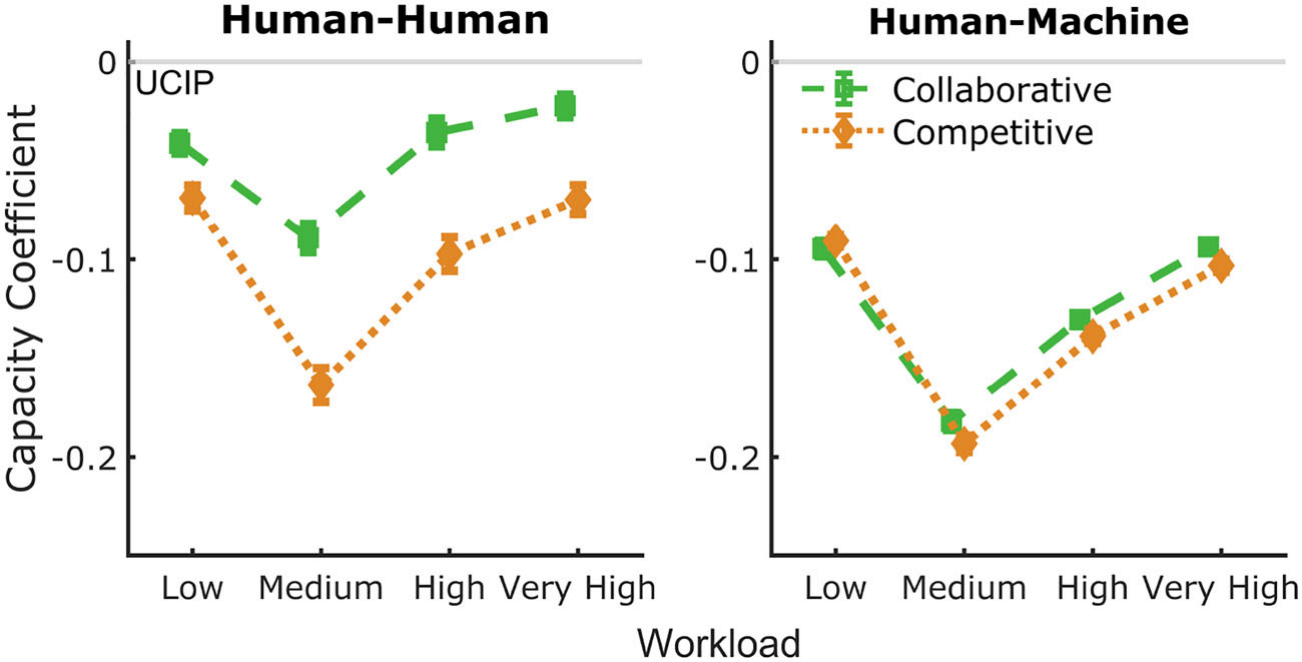
Aggregated capacity coefficients for all workload levels in the collaborative (green) and competitive (orange) groups for HH (left) and HM (right) teams. The UCIP benchmark (gray) was determined for each dyad by taking the product of the dyad member scores in the separate group condition. Capacity coefficients were below the UCIP benchmark for all conditions. Collaborative teams returned greater C(p) values than competitive teams in the HH task. This trend was reduced in HM teams.

**Fig. 6:**
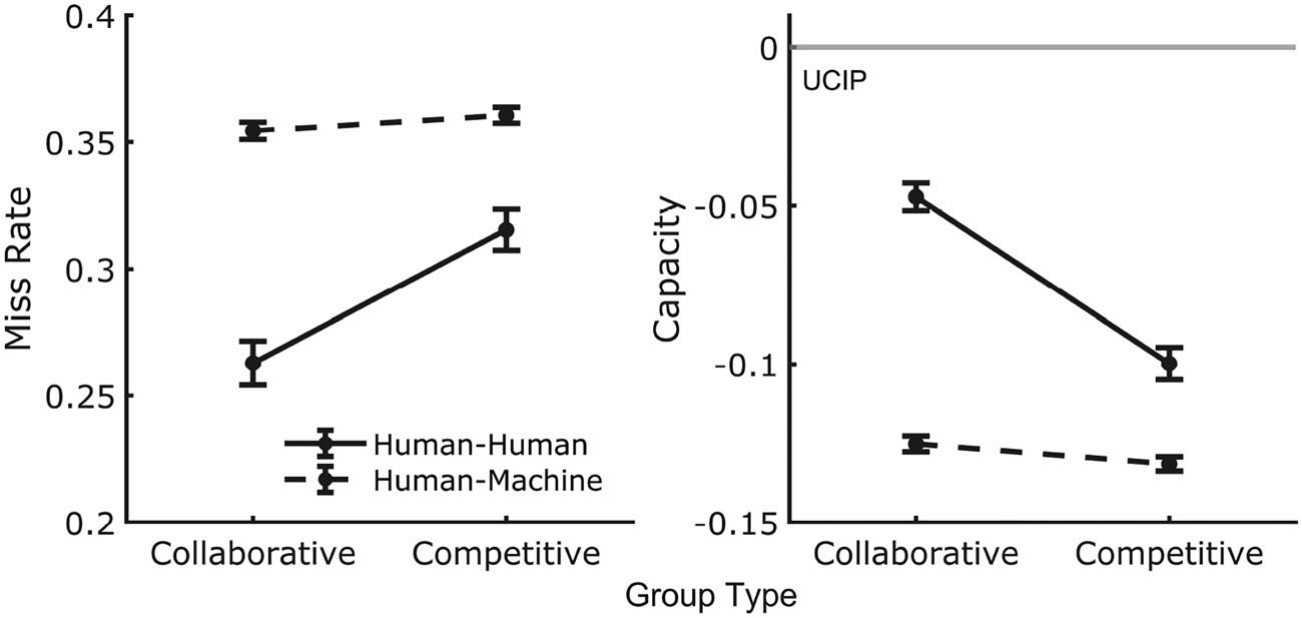
Post hoc examination of interaction effect in miss rates (left) and workload capacity (right) between collaborative and competitive groups in HH and HM teams.

#### 3.1.2 *n* Balls maintained

Player, machine agent, and team miss rates were then used to estimate the number of balls maintained with a 40% miss rate, with the density of converted scores shown below in Fig. 4. Although many of the relationships in this section align with those reported as miss rates, this section is included to present an intuitive indication of the effects of different team conditions.

The number of balls maintained was assessed using a mixed‐effects anova with repeated measures of group condition (collaborative, competitive, player) and between‐subjects factor of team type (human or machine). We found a significant main effect of group condition ($F_{\left(1.87,651.36\right)}=336.48$, p<.001, $BF_{inclusion}>1,000$), team‐type ($F_{\left(1,347\right)}=64.22$, p<.001, $BF_{inclusion}>1,000$), and the interaction between these factors ($F_{\left(1.88,651.36\right)}=86.22$, p<.001, $BF_{inclusion}>1,000$).

Participants in HH teams were able to maintain an average of 1.24 more balls than players in the HM condition (t=8.01, p<.001, posterior odds >1,000). Collaborative and competitive groups were able to maintain a significantly greater amount of balls than individuals (collaborative: +1.91, t=25.59, p<.001, posterior odds >1,000; competitive: +1.23, t=16.47, p<.001, posterior odds >1,000). Collaborative groups produced an additional average of 1.22 balls than collaborative groups in the HH teams (t=9.12, p<.001, posterior odds >1,000). However, there was no significant performance benefit observed when collaborating versus competing in an HM team (+0.14 balls, t=2.21, p=.408, posterior odds =1.87).

Finally, at a simple descriptive level, collaborative and competitive group performance was greater than the separate condition but still below the UCIP benchmark for HH and HM teams. This simple transformation provides a tangible interpretation: collaborative HH teams can generate greater team performances than individuals. However, this greater collaborative performance is still 1.03 *fewer* balls (i.e., a cost) than the performance we would theoretically expect from combining two independently working individuals.

#### 3.1.3 Workload capacity

Mean capacity coefficient scores across workload and group conditions are shown in Fig. 5 for both team types, with reference to the UCIP benchmark at C(p) = 0. All capacity coefficient values were below the UCIP benchmark, indicating limited workload capacity under collaborative and competitive group conditions for HH and HM teams. This result indicates that teams returned better miss rates than individuals but teamwork resulted in *costs* to the overall group performance. We conducted a 2×4×2 mixed anova with team type as the between‐subjects factor, with two group conditions (collaborative, competitive) and all workload levels as repeated measures.

Again, all main‐ and interaction effects were significant (team‐type: $F_{\left(1,349\right)}=126.37$, p<.001, $BF_{inclusion}>1,000$; group condition: $F_{\left(1,349\right)}=133.33$, p<.001, $BF_{inclusion}>1,000$; workload: $F_{\left(2.86,999.49\right)}=168.5$, p<.001, $BF_{inclusion}>1,000$), with HH teams recording a higher capacity coefficient than HM teams (mean difference = 0.055, *t* = 11.24, *p* <  .001).

Capacity coefficients of collaborative teams were significantly higher than competitive teams (mean difference = 0.006, t=11.55, p<.001, posterior odds >1,000). Post hoc analysis of the interaction indicated that HH teams produced higher capacity coefficients than HM teams in both collaborative and competitive teams (*p* < .001), and both HH and HM teams returned higher collaborative coefficients than competitive teams (all *p* < .001 except machine‐collaborative vs. machine‐competitive: *p* = .023, posterior odds = 25.15).

We again examined the interaction between team‐type and collaborative and competitive group conditions via the capacity data by collapsing across workload (see Fig. 6, right). The assessment of workload capacity scores is particularly beneficial here as data for each dyad's performance has been scaled by the performance of the team's members, thereby reducing measurement variance within each team. We found a significant interaction with extreme evidence ($F_{\left(1,349\right)}=82.5$, p<.001, $BF_{inclusion}>1,000$).

Teams produced the highest capacity coefficients in the low and very high workload conditions, with the difference between the two levels not significantly different and strong evidence that the lowest and highest workload levels were the same (t=−0.369, p=1, posterior odds = 0.035). Teams in the high workload level produced the next highest coefficients, significantly lower than the low and very high levels but significantly higher than the lowest capacity values observed in the medium workload level (all *p* < .001, posterior odds > 1,000). However, a specific examination of HH teams indicated that collaborative and competitive groups in the medium workload produced the lowest capacity values but the low, high, and very high workload conditions differed non‐significantly (*p* > .05).

### 3.2 Behavioral patterns

#### 3.2.1 Momentary distance

**Fig. 7:**
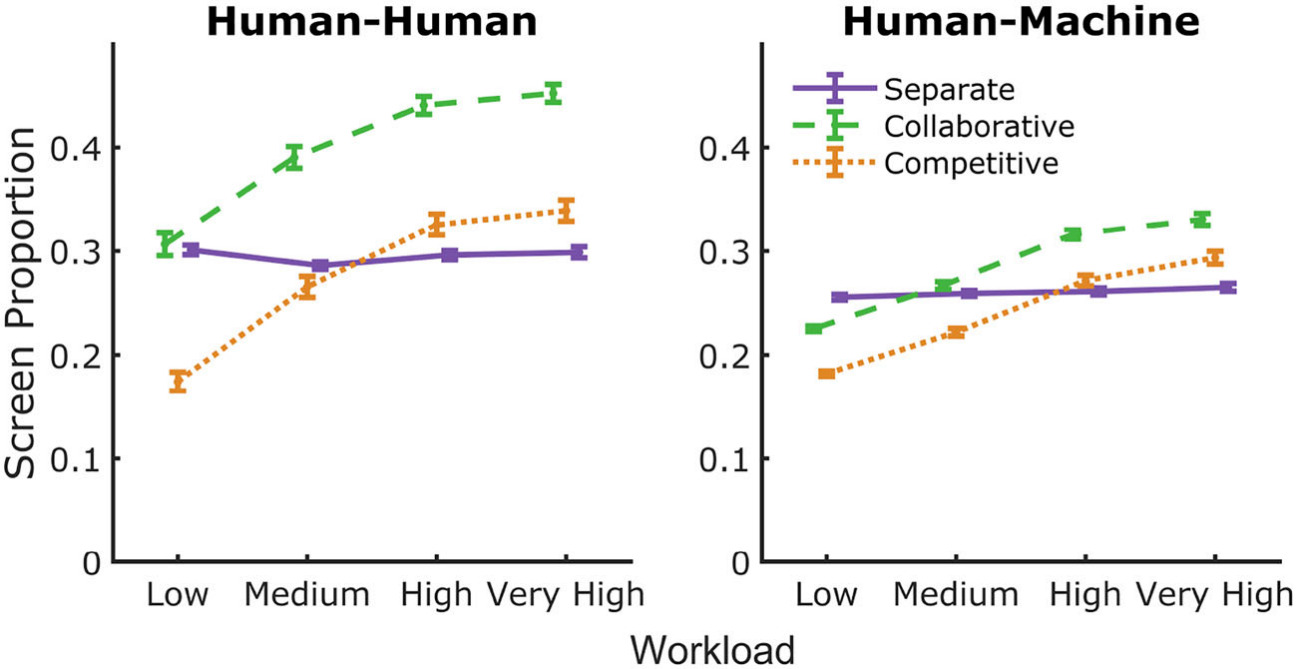
Momentary distances in HH (left) and HM teams (right). Distance between players in team conditions increased with workload was highest in collaborative groups and was reduced in competitive groups. Distances between paddles in the separate team condition offer a helpful marker for team strategy when player action is independent of the other.

Momentary distance means are shown in Fig. 7. We found significant main effects of (i) team type ($F_{\left(1,349\right)}=67.90$, p<.001, $BF_{inclusion}>1,000$), where HM teams displayed a lower paddle separation than HH teams (t=−6.65, p<.001, posterior odds >1,000); (ii) group ($F_{\left(1.92,668.32\right)}=353.90$, p<.001, $BF_{inclusion}>1,000$), where collaborative groups divided the workspace more evenly than competitive groups (t=−4.26, p<.001, posterior odds >1,000); and (iii) workload ($F_{\left(1.77,618.78\right)}=409.29$, p<.001, $BF_{inclusion}>1,000$), where paddle separation increased in collaborative and competitive groups as the workload increased (all p<.001, posterior odds > 1, 000) up to the high load condition where separation plateaued.

#### 3.2.2 Highly correlated segments

**Fig. 8:**
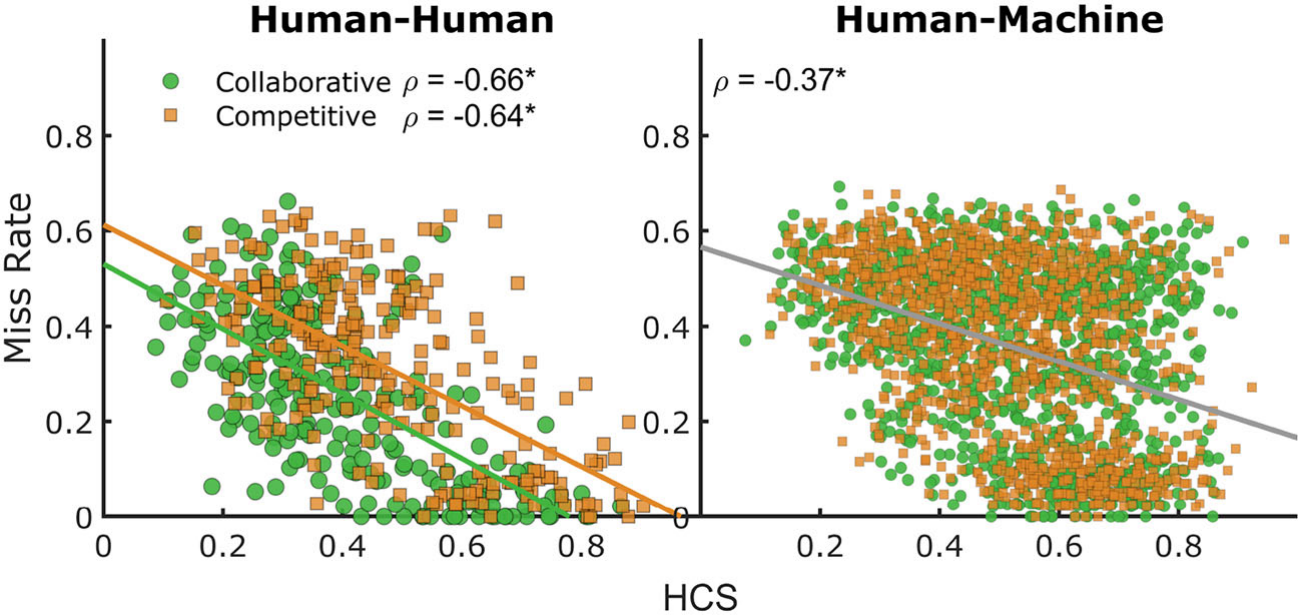
Correlation between miss rate and HCS in collaborative and competitive teams. Collaborative HH teams have better miss rates and lower HCS than competitive HH teams, a trend absent in HM teams. * indicates significance at *p* < .05.

We correlated the proportion of HCS with miss rates to examine the relationship between correlated behavior and performance between collaborative and competitive dyads (Fig. 8). The proportion of time spent in highly correlated movements was negatively correlated in HH (ρ=0.66,p<.001) and HM teams (ρ=0.57,p<.001). However, correlated behavior in HH teams was lower overall in collaboration compared to competitive groups (mean difference = −0.085, t=−6.6, p<.001, posterior odds >1,000) but coordinated action was similar across team types in HM teams.

## 4 Discussion

Our study utilized a novel, dynamic, two‐player team‐based task to examine human performance in HM and HH teams. To evaluate the effects of different intragroup interactions on group performance, we manipulated group composition (HH and HM groups), group social conditions (collaborative, competitive, and separate), and the level of workload (four levels, from low to very high). To summarize the key findings, our three hypotheses were supported: Primary task performance was greater in human groups than HM teams; collaborative group conditions resulted in greater primary task performance than competitive groups, and both group conditions outperformed participants completing the task separately; and there was a decrease in primary task performance as the workload increased for all group types and team compositions.

To quantify group efficiency, we applied workload capacity analysis which indicated performance costs in the group setting across conditions, although not so large that individual performance was lower than group performance in primary task measures. That is, groups produced greater performance, but this performance was *less* than the output expected from two independently working individuals. Further, for HH teams, there was less cost in the collaborative condition than in the competitive condition. Interestingly, we did not observe the reduced cost across conditions in the HM teams.

Through examining the behavioral patterns, we found collaborative groups adopted spatial‐sharing strategies, whereas participants in competitive group conditions tracked their opponents' positions. These strategies were similar across HH and HM teams but were muted in HM teams. Further, we found a significant negative correlation between the level of interactions between group members and the costs to group performance. Concretely, group performance costs decreased (performance improved) when the level of within‐trial correlated behaviors decreased.

We propose that these results reflect the inability of HM groups to incorporate or act on relevant team strategy information. Overall, our implementation of workload capacity analysis in the HM group performance domain presents a robust methodological and assessment framework for quantifying and evaluating HM teams and furthers our understanding of the group conditions conducive to collective HM intelligence. The following section discusses the efficacy and implications of workload capacity analysis for evaluating HM team performance. We then discuss the features of collaborative and competitive conditions on HM group performance in our task before discussing our findings from the behavioral pattern analyses and how the underlying behavioral processes of a group can inform sources of group performance costs.

### 4.1 Human‐machine team efficiency

We found that human and HM teams performed better than participants completing the task individually in the separate condition. This result indicates a performance benefit of completing the task in groups. However, workload capacity analysis showed that the group performance capacity was limited in human and HM teams and collaborative and competitive group conditions. Interestingly, collaborative human groups reduced this group performance cost. This result indicates that, while HM teams can outperform individuals, collaborating HM dyads in our task experienced greater limitations from inefficient group interaction processes.

Workload capacity analysis provides a quantitative assessment of group performance by scaling the group performance against the combined performances of the group members when working alone. In our task, this translates to the collaborative or competitive group miss rates for each dyad being scaled by the performance of each dyad's members in the separate group condition. This process yields the capacity coefficient measure, which is compared against the UCIP benchmark. The UCIP benchmark is a theoretical benchmark representing the expected performance had the two group members completed the task simultaneously and independently. That is, without interacting in any way. Comparison of group performance to this benchmark provides compelling theoretical implications. As collaborative and competitive group performance on our task was limited across both team composition types, this presents two simple conclusions: (i) team members exchange information to complete the task and (ii) these exchanges limit the team's potential output.

The group performance costs described here can also be understood in terms of ‘process loss’ (Steiner, 1966). Teams have a maximum potential level of productivity, but inefficient or faulty processes within the group can reduce the group output. The difference between a group's potential output and actual output indicates costs associated with inefficient group processes. The application of workload capacity as both a measure of team performance and an indicator of efficient group processes will benefit future research aiming to objectively quantify and assess the process losses or process gains (Mejias, 2007; Weber & Hertel, 2007) that may emerge under different HM group conditions.

Additionally, as workload capacity analysis can characterize the team conditions resulting in performance costs or gains, specific features of machine agent design can be targeted or optimized. Of particular interest to the current research and a promising channel for further examination was the statistical interaction between group conditions and team types. Specifically, the costs of team performance were reduced in collaborative teams compared to competitive teams but only for HM dyads; these collaborative benefits were *not* observed for HM teams (see Fig. 6). We discuss the features of human and HM teams in our task that may have led to this outcome.

### 4.2 Collaborative and competitive human‐machine teams

Information‐exchange processes naturally exist between HH team members, leading to more efficient collaborative behavior. Both team members can actively engage in team‐critical tasks, such as coordinating actions or providing support between members. The reinforcement learning and ideal‐observer‐simple machine agent algorithms used in our task could be indirectly affected by (and affect) the human player since they are responding to common signals. For example, the human‐controlled paddle may deflect a ball in a way that sends the ball to the right side of the screen, encouraging the bot to move toward the predicted trajectory. The bot's behavior could have differed if the participant had missed the ball or hit from a different angle. On the other hand, we programmed the “considerate” iteration of the ideal‐observer algorithm to avoid the human participants position during the collaborative condition directly (see the Appendix). While the considerate agent produced responsive behaviors designed for collaboration with the human participant, these behavioral properties were insufficient to produce meaningful differences in HM performance. Therefore, the group interactions between collaborating humans did not appear to emerge naturally from the human participants in the HM dyads. Future work can build more sophisticated team awareness in the machine agents. For example, although advancing machine agent complexity is not necessary for improved task performance (Bansal et al., 2019), implementing greater complexity of collaborative behaviors or the responsiveness of machine agents to human collaborators via adaptive performance mechanisms (e.g., Zhao et al., 2022) may help reduce the group performance costs in our task. In any case, workload capacity analysis represents a practical tool for exploring different machine agent mechanisms and designs in future research and applications.

Finally, the extent to which collaborative and competitive group conditions were established by the task design may also influence the group performance outcomes. For example, the group conditions in our task were generated via instructions preceding each experimental block. Without additional reinforcement of the group conditions throughout the block, such as financial incentives to perform well, group performance costs may emerge from weak group manipulations rather than inefficient group interactions. Furthermore, manipulations of the collaborative group type may have been limited by the presentation of individual player scores throughout trials. Such details in task design can affect participant behavior differently. For example, highlighting individual contributions to the group can encourage participants to focus on their scores. However, it can also increase the accountability of individuals to the group, thereby encouraging active group engagement (Hardy & Latané, 1986). Additionally, research on bidding behaviors has shown that competing participants exhibit greater engagement and joy from winning bids against human competitors than a machine agent (Teubner, Adam, & Riordan, 2015). Therefore, the increased costs to the performance observed in HM teams may stem from the human group member limiting their engagement with the machine agent. While task and agent design limitations may help inform potential causes of group performance costs, previous research has connected limited capacity with the inefficient processing of behavioral cues between team members. Our preliminary analysis of behavioral patterns can inform the extent to which participants engaged in interactive group behaviors specific to collaboration and competition and, therefore, the sources of group performance costs.

### 4.3 Behavioral pattern analysis

Previous research indicating performance costs in collaborative human teams by Yamani et al. (2017) found a correlation between C(p) values of dyads and their overlapping scan patterns in a visual search task. The authors credited limited capacity to increased gaze‐linking between individuals. Similarly, our preliminary analysis of behavioral patterns coincided with limited capacity performance costs teams and indicated clear distinctions in participant performance strategies under our collaborative and competitive group manipulations.

We examined the momentary distance between players and the proportion of HCS to indicate player behavior and team strategy. We found collaborative groups divided the workspace more evenly and displayed a lower proportion of correlated behaviors, whereas competitive players had smaller interpaddle distances and higher correlated behavior. Our assessment of momentary distances indicates a screen‐sharing strategy in collaborative dyads, where participants divide the playing space to distribute the workload among group members, and a monitoring strategy in competitive dyads, where players operate within similar spaces to compete for targets. The HCS results support this intuition; collaborative dyads displayed lower HCS values, indicating that collaborators first separated their playing space and then operated somewhat independently of their teammate within that playing space. In contrast, competitive participants displayed higher HCS values, indicating a greater proportion of trial time where participant actions were made in response to changes made by their opponent. Simple correlation analysis indicated that these behavioral patterns correspond with the observed differences in task performance (see Fig. 8).

Group performance costs of collaboration we observed in HH teams may occur when collaborators do not assist struggling teammates or if assisting overloads the collaborator. However, although a cost prevails in collaborative teams, the screen‐sharing strategy and lower team‐member motion correlations coincided with reduced team workload costs compared to competitive teams. Although machine agents in this task were designed to reproduce human‐like performance outcomes, machine agent actions lacked the global contextual cues processed by humans, a common issue in HM research (e.g., McNeese, Demir, Cooke, & She, 2021). Specifically, machine performance in our task depended entirely on ball‐related information, which neglects critical information typically utilized by a human agent. For example, human players may incorporate the spatial relationships between targets and team members according to the current group conditions (i.e., collaboration) as the machine agent has no understanding of the team strategy or the ability to update decisions based on teammate behavior (i.e., the machine is not a participant in the team's collective intelligence), the efficacy of the strategy degrades, leading the costs of teamwork to increase in these HM teams.

The results from our behavioral analysis might also indicate that participants in HM teams were less willing to engage in team‐based interactions, such as collaboration. Although distinct spatial sharing strategies were evident in HM dyads, these strategies were reduced compared to collaborative human dyads. It is possible that human participants did not perceive the machine agents as responsive to their actions, thereby reducing their collaborative behaviors and limiting the performance benefits associated with collaborative strategies. The utility of workload capacity as a team performance measurement device becomes clear here, where poor team or task conditions can be identified and potentially rectified. For example, via comparison to HH collaborative teams, we can infer that HM teams in our task may suffer as both team members were unable to share and interpret team‐relevant information, such as task strategy.

### 4.4 Future work and conclusions

Future research can examine the effects of more complex collaborative and competitive group interactions. For example, a collaborative condition where the workload between group members is manipulated such that one group member is overloaded and requires assistance from group members may increase the costs of group performance. Similarly, a competitive task where player paddles cannot cross increases the influence of competitive strategies. Such research would complement the findings from the current experimental design, where group performance was low in interdependence. That is, the group output in our task was the result of adding the individual contributions of each group member (Forsyth, 2018; Steiner, 1972) and the complexity of group interactions, such as collaboration, was low. A preliminary indication of the effects of task complexity on group processes is visible in the non‐linear changes to workload capacity outcomes across workload levels. That is, costs to group performance for all team types increased in the medium workload condition but were similar in the low, high, and very high conditions. Utilizing the complementary results from our behavioral analyses, we conclude that participants may use global strategies for the task (i.e., screen sharing or monitoring), and the participants may alter their performance strategies to manage changes in task complexity (i.e., workload).

Finally, future investigations into the role of COHUMAIN should explore differences between HH teams and HM teams in which the machine agents possess the ability to engage in behaviors that assist the emergence of collective intelligence. In our findings, HH teams demonstrated the ability to incorporate behavioral cues to adjust their behavior, resulting in less correlated behavior and a spatial division strategy under collaborative conditions. These behaviors indicate a shared task understanding and a shared understanding of the roles of each group member. Critically, we found greater group performance related to these collaborative group interactions, but HM teams failed to produce this benefit. Based on these findings, teams in which machine agents can produce a global performance strategy and update actions according to this strategy may demonstrate similar behavior and performance efficiency to human teams.

Our methodology and design present a unique and novel approach for examining and assessing HM team performance and the group interaction processes utilized under various team and group conditions to produce a final team‐level output. Critically, our application of workload capacity analysis within HM teams provides researchers with a pragmatic tool to assess the efficiency and quality of group‐member integration within HM team combinations. Our study specifically assessed the effects of collaboration and competition. However, the measurement framework is readily extended to other HM environments, a timely inclusion for research and exploration of collective HM intelligence.

## Supporting information



Supplementary Material

## Data Availability

Data, experiment code, and analyses for the present study are available online at https://osf.io/mtazf.

## Appendix A Agent design

### A.1. Ideal observer

Concretely, location weights are calculated for each ball by evaluating an *x* coordinate of where the ball will next intersect the paddle line and the time (*t*) in seconds before the intersection occurs is calculated. These calculations consider deflections that are or will be occurring throughout the game area before the intersection. Each of the 80 locations can be weighted according to temporal and spatial proximity to each ball. If a large number of balls will intersect the paddle line close to a particular location (close spatial proximity), not too far into the future (close spatial proximity), then such a location would be weighted more highly than an impact occurring physically or temporally further away.

Balls with *t*‐coordinates more than 1.2 s into the future were not considered to restrict performance and emulate human performance. Note that the considerate and simple agents are identical, except that the considerate agent sets all locations within 100 pixels of the participant's paddle to zero during the collaborative team condition.

### A.2. Reinforcement learning

The quality of an action is a simple numerical representation updated on each screen update during training only and is given by

(A.1)
$$Q^{new}\left(s_{t},a_{t}\right)\leftarrow \left(1-\alpha \right)\cdot Q\left(s_{t},a_{t}\right)+\alpha \cdot \left(r_{t}+\gamma \cdot \max Q_{a}\left(s_{t+1},a\right)\right),$$

where the updated quality ($Q^{new}$) for a given state (s) and action (a) at the current time (t) is determined by the reward (r) for the current action, mediated by the learning rate (α), future rewards (the maximum *q*‐value at the next time step), and a discount factor for future rewards (γ).

In our experiment, the state is the relative position of the paddle to a ball and the actions are to move left, right, or remain stationary. We determine the state space by dividing the screen into a 40×20 grid and then determining the relative distance between the paddle and the ball according to these spaces. Each row and column of the grid represents the relative distance between the paddle and the ball and contains the quality values for each action given the relative position.

The agent was rewarded for hit events with a score of +50 and rewarded at each step according to the *x*‐coordinate difference between the ball and paddle, multiplied by the *y*‐coordinate difference. This reward structure increases the value of movements that reduce the *x*‐distance between the paddle and the ball with a greater weight applied to the reward as the ball nears the paddle intercept point.

The agent selected for this experiment was trained over 40,000 episodes. An episode involved a single ball randomly positioned along the top of the screen set to a random trajectory and the paddle initiated at a random position along the bottom of the screen and concluded when the ball was either hit or missed. Paddle movements throughout training were determined by the maximum *q*‐value if a randomness threshold, ε, was met or selected randomly. Initial *q*‐values were generated randomly, but other training parameters were modified to achieve a mean miss‐rate of approximately 0.05, similar to human performance in the single ball condition: a learning rate of 0.1, a discount factor of 0.99, ε was initialized at 1 and decayed at the completion of each episode at a rate of 0.9999.

## References

[tops12683-bib-0001] Adam, M. T. , Eidels, A. , Lux, E. , & Teubner, T. (2017). Bidding behavior in Dutch auctions: Insights from a structured literature review. International Journal of Electronic Commerce, 21(3), 363–397.

[tops12683-bib-0002] Algom, D. , Eidels, A. , Hawkins, R. X. , Jefferson, B. , & Townsend, J. T. (2015). Features of response times: Identification of cognitive mechanisms through mathematical modeling. In J. R. Busemeyer , Z. Wang , J. T. Townsend , & A. Eidels (Eds.), The Oxford handbook of computational and mathematical psychology (pp. 63–98). Oxford, England: Oxford University Press.

[tops12683-bib-0003] Bansal, G. , Nushi, B. , Kamar, E. , Weld, D. S. , Lasecki, W. S. , & Horvitz, E. (2019). Updates in human‐AI teams: Understanding and addressing the performance/compatibility tradeoff. In Proceedings of the AAAI conference on artificial intelligence (Vol. 33, pp. 2429–2437). Washington, DC: AAAI Press.

[tops12683-bib-0004] Bennett, M. , Mullard, R. , Adam, M. T. , Steyvers, M. , Brown, S. , & Eidels, A. (2020). Going, going, gone: Competitive decision‐making in Dutch auctions. Cognitive Research: Principles and Implications, 5(1), 1–22. 33252772 10.1186/s41235-020-00259-w PMC7704846

[tops12683-bib-0005] Brennan, A. A. , & Enns, J. T. (2015a). What's in a friendship? Partner visibility supports cognitive collaboration between friends. PloS ONE, 10(11), e0143469. 26619079 10.1371/journal.pone.0143469 PMC4664270

[tops12683-bib-0006] Brennan, A. A. , & Enns, J. T. (2015b). When two heads are better than one: Interactive versus independent benefits of collaborative cognition. Psychonomic Bulletin & Review, 22(4), 1076–1082. 25416077 10.3758/s13423-014-0765-4

[tops12683-bib-0007] Brennan, S. E. , Chen, X. , Dickinson, C. A. , Neider, M. B. , & Zelinsky, G. J. (2008). Coordinating cognition: The costs and benefits of shared gaze during collaborative search. Cognition, 106(3), 1465–1477. 17617394 10.1016/j.cognition.2007.05.012

[tops12683-bib-0008] Camerer, C. F. , & Hogarth, R. M. (1999). The effects of financial incentives in experiments: A review and capital‐labor‐production framework. Journal of Risk and Uncertainty, 19(1), 7–42.

[tops12683-bib-0009] Correia, F. , Melo, F. S. , & Paiva, A. (2022). When a robot is your teammate. *Topics in Cognitive Science*. Advance online publication. 10.1111/tops.12634 36573665

[tops12683-bib-0010] Demir, M. , McNeese, N. J. , & Cooke, N. J. (2017). Team situation awareness within the context of human‐autonomy teaming. Cognitive Systems Research, 46, 3–12.

[tops12683-bib-0011] Eidels, A. , & Gold, J. (2014). Measuring single‐item identification efficiencies for letters and 3‐D objects. Behavior Research Methods, 46(3), 722–731. 24254880 10.3758/s13428-013-0417-z

[tops12683-bib-0012] Eidels, A. , Townsend, J. T. , Hughes, H. C. , & Perry, L. A. (2015). Evaluating perceptual integration: Uniting response‐time‐and accuracy‐based methodologies. Attention, Perception, & Psychophysics, 77(2), 659–680. 10.3758/s13414-014-0788-y 25366823

[tops12683-bib-0013] Forsyth, D. R. (2018). Group dynamics. Boston, MA: Cengage Learning.

[tops12683-bib-0014] Geisler, W. S. (2003). Ideal observer analysis. The Visual Neurosciences, 10(7), 12–12.

[tops12683-bib-0015] Grimm, D. A. , Demir, M. , Gorman, J. C. , & Cooke, N. J. (2018). Team situation awareness in human‐autonomy teaming: A systems level approach. In Proceedings of the Human Factors and Ergonomics Society annual meeting (Vol. 62, pp. 149–149). Los Angeles, CA: Sage.

[tops12683-bib-0016] Gupta, P. , & Woolley, A. W. (2021). Articulating the role of artificial intelligence in collective intelligence: A transactive systems framework. In Proceedings of the Human Factors and Ergonomics Society annual meeting (Vol. 65, pp. 670–674). Los Angeles, CA: Sage.

[tops12683-bib-0017] Hardy, C. , & Latané, B. (1986). Social loafing on a cheering task. Social Science, 71(2–3), 165–172.

[tops12683-bib-0018] Heathcote, A. , Coleman, J. R. , Eidels, A. , Watson, J. M. , Houpt, J. , & Strayer, D. L. (2015). Working memory's workload capacity. Memory & Cognition, 43(7), 973–989. 25962602 10.3758/s13421-015-0526-2

[tops12683-bib-0019] Hedley, L. , Bennett, M. S. , Love, J. , Houpt, J. W. , Brown, S. D. , & Eidels, A. (2023). The relationship between teaming behaviours and joint capacity of hybrid human‐machine teams. In *Proceedings of the Cognitive Science Society Conference*. Austin, TX: Cognitive Science Society.

[tops12683-bib-0020] Hsieh, C.‐J. , Fifić, M. , & Yang, C.‐T. (2020). A new measure of group decision‐making efficiency. Cognitive Research: Principles and Implications, 5(1), 1–23. 32940791 10.1186/s41235-020-00244-3 PMC7498531

[tops12683-bib-0021] Marcelino, R. , Sampaio, J. , Amichay, G. , Gonçalves, B. , Couzin, I. D. , & Nagy, M. (2020). Collective movement analysis reveals coordination tactics of team players in football matches. Chaos, Solitons & Fractals, 138, 109831.

[tops12683-bib-0022] McNeese, N. J. , Demir, M. , Chiou, E. K. , & Cooke, N. J. (2021). Trust and team performance in human–autonomy teaming. International Journal of Electronic Commerce, 25(1), 51–72.

[tops12683-bib-0023] McNeese, N. J. , Demir, M. , Cooke, N. J. , & She, M. (2021). Team situation awareness and conflict: A study of human–machine teaming. Journal of Cognitive Engineering and Decision Making, 15(2–3), 83–96.

[tops12683-bib-0024] Mejias, R. J. (2007). The interaction of process losses, process gains, and meeting satisfaction within technology‐supported environments. Small Group Research, 38(1), 156–194.

[tops12683-bib-0025] National Academies of Sciences, Engineering, & Medicine (2022). Human‐AI teaming: State‐of‐the‐art and research needs. Washington, DC: The National Academies Press.

[tops12683-bib-0026] Niehorster, D. C. , Cornelissen, T. , Holmqvist, K. , & Hooge, I. (2019). Searching with and against each other: Spatiotemporal coordination of visual search behavior in collaborative and competitive settings. Attention, Perception, & Psychophysics, 81(3), 666–683. 10.3758/s13414-018-01640-0 PMC6407732 30593653

[tops12683-bib-0027] Scott‐Sharoni, S. T. , Yamani, Y. , Kneeland, C. M. , Long, S. K. , Chen, J. , & Houpt, J. W. (2021). Exploring the effects of perceptual separability on human‐automation team efficiency. Computational Brain & Behavior, 4(4), 486–496.

[tops12683-bib-0028] Steiner, I. D. (1966). Models for inferring relationships between group size and potential group productivity. Behavioral Science, 11(4), 273–283. 5945374 10.1002/bs.3830110404

[tops12683-bib-0029] Steiner, I. D. (1972). Group process and productivity. New York, NY: Academic Press.

[tops12683-bib-0030] Surowiecki, J. (2005). The wisdom of crowds. New York, NY: Anchor.

[tops12683-bib-0031] Teubner, T. , Adam, M. , & Riordan, R. (2015). The impact of computerized agents on immediate emotions, overall arousal and bidding behavior in electronic auctions. Journal of the Association for Information Systems, 16(10), 838.

[tops12683-bib-0032] Townsend, J. T. , & Eidels, A. (2011). Workload capacity spaces: A unified methodology for response time measures of efficiency as workload is varied. Psychonomic Bulletin & Review, 18(4), 659–681. 21607804 10.3758/s13423-011-0106-9

[tops12683-bib-0033] Townsend, J. T. , & Nozawa, G. (1995). Spatio‐temporal properties of elementary perception: An investigation of parallel, serial, and coactive theories. Journal of Mathematical Psychology, 39(4), 321–359.

[tops12683-bib-0034] Walliser, J. C. , de Visser, E. J. , Wiese, E. , & Shaw, T. H. (2019). Team structure and team building improve human–machine teaming with autonomous agents. Journal of Cognitive Engineering and Decision Making, 13(4), 258–278.

[tops12683-bib-0035] Watkins, C. J. , & Dayan, P. (1992). Q‐learning. Machine Learning, 8(3), 279–292.

[tops12683-bib-0036] Weber, B. , & Hertel, G. (2007). Motivation gains of inferior group members: a meta‐analytical review. Journal of Personality and Social Psychology, 93(6), 973. 18072849 10.1037/0022-3514.93.6.973

[tops12683-bib-0037] Wichmann, F. A. , & Hill, N. J. (2001). The psychometric function: I. Fitting, sampling, and goodness of fit. Perception & Psychophysics, 63(8), 1293–1313. 11800458 10.3758/bf03194544

[tops12683-bib-0038] Woolley, A. W. , Chabris, C. F. , Pentland, A. , Hashmi, N. , & Malone, T. W. (2010). Evidence for a collective intelligence factor in the performance of human groups. Science, 330(6004), 686–688. 20929725 10.1126/science.1193147

[tops12683-bib-0039] Yamani, Y. , & McCarley, J. S. (2016). Workload capacity: A response time–based measure of automation dependence. Human Factors, 58(3), 462–471. 26811351 10.1177/0018720815621172

[tops12683-bib-0040] Yamani, Y. , & McCarley, J. S. (2018). Effects of task difficulty and display format on automation usage strategy: A workload capacity analysis. Human Factors, 60(4), 527–537. 29470135 10.1177/0018720818759356

[tops12683-bib-0041] Yamani, Y. , Neider, M. B. , Kramer, A. F. , & McCarley, J. S. (2017). Characterizing the efficiency of collaborative visual search with systems factorial technology. Archives of Scientific Psychology, 5(1), 1.

[tops12683-bib-0042] Zajonc, R. B. (1965). Social facilitation: A solution is suggested for an old unresolved social psychological problem. Science, 149(3681), 269–274. 14300526 10.1126/science.149.3681.269

[tops12683-bib-0043] Zhao, M. , Simmons, R. , & Admoni, H. (2022). The role of adaptation in collective human–AI teaming. Topics in Cognitive Science, 17(2), 291–323. 10.1111/tops.12633 PMC12093936 36374986

[tops12683-bib-0044] Zinn, C. M. , Yamani, Y. , Houpt, J. W. , & Scott‐Sharoni, S. (2018). Assessment function analysis of human‐automation team performance: A reanalysis of data from Yamani and Mccarley (2018). In Proceedings of the Human Factors and Ergonomics Society annual meeting (Vol. 62, pp. 1540–1544). Los Angeles, CA: Sage.

