# Supplementary material for: Human Performance in Competitive and Collaborative Human–Machine Teams
Source: Top Cogn Sci. 2023 Jul 13;17(2):324–48. doi: 10.1111/tops.12683 (PMC12093930; doi:10.1111/tops.12683)
Supplement: Supplementary file 1 — Supplementary Material [file TOPS-17-324-s001.pdf]

Supplementary Material

Cognitive Load in Human-Machine Teams

We first distinguish here between our use of the terms "workload capacity" and "cognitive load", as workload capacity is often used as a measure of cognitive load. However, *Workload capacity* is used in the current article to refer to the measurement of efficient *team* performance. *Cognitive load* is a broad term that refers to the mental resources an individual needs to complete a task (Thorpe, Nesbitt, & Eidels, 2020). The relationship between performance and an *individual's* available cognitive resources is well documented (Innes, Howard, Thorpe, Eidels, & Brown, 2021; Thorpe et al., 2020) where the cognitive load experienced by an individual will typically increase under greater task demands, leading to decreased performance (Wickens, 2002). There is an interesting trade-off when considering cognitive load within team settings: completing tasks in a team can alleviate task load for individuals, thereby reducing cognitive load. However, team-related tasks might also increase cognitive load (Bowers, Braun, & Morgan, 1997; Funke, Knott, Salas, Pavlas, & Strang, 2012).

The cognitive load experienced by individuals within teams is typically assessed using subjective self-reports (Funke et al., 2012) adapted from measures validated for the assessment of individuals. This methodology provides rapid assessment and allows qualitative access to perceived sources of cognitive load (e.g., communication, team-member capability). However, self-report questionnaires lack temporal specificity and objectivity. These two critical cognitive load measurement issues can be resolved using a concurrent secondary task (Howard, Innes, Eidels, & Loft, 2021; Strayer, Cooper, Turrill, Coleman, & Hopman, 2015, e.g.,). Performance on a secondary task, such as the detection response task (DRT; see `https://osf.io/mtazf/`), indexes the cognitive resources remaining from the primary task. As the two tasks are performed concurrently, cognitive load can be measured objectively *within* the task.

We embedded the Detection Response Task as a secondary task within the design of the current experiment to quantitatively assess the effects of group composition and conditions on cognitive performance. We include the analysis and results from this

experiment online at `https://osf.io/mtazf/`, but present a brief outline of the key findings here.

We found no difference in cognitive load measures between team compositions but observed faster response times and higher accuracy in individuals when completing the task separately compared to the collaborative and competitive team conditions. Participants of competitive teams returned faster response times than collaborative teams. We encourage examining these different team processes for future work but suggest that our results indicated a cognitive cost of teamwork processes (Funke et al., 2012) and a greater cognitive resource requirement of collaborative teams. However, this effect was only apparent in the low workload condition.

While cognitive load was unaffected by team composition, the DRT was sensitive to workload levels and group conditions, albeit constrained by the difficulty of our task. As such, future examinations of more advanced human-machine combinations (e.g., AI capable of interpreting or dynamically updating behaviour according to team strategy) are encouraged to continue the empirical, objective measurement of cognitive load experienced by human participants in machine teams via the DRT. Doing so will allow researchers to understand better the cognitive demand of various human-machine designs and team interactions.

References

Bowers, C. A., Braun, C. C., & Morgan, B. B. (1997). *Team workload: Its meaning and measurement.* Psychology Press.

Funke, G. J., Knott, B. A., Salas, E., Pavlas, D., & Strang, A. J. (2012). Conceptualization and measurement of team workload: A critical need. *Human Factors*, *54*(1), 36–51.

Howard, Z. L., Innes, R., Eidels, A., & Loft, S. (2021). Using past and present indicators of human workload to explain variance in human performance. *Psychonomic Bulletin & Review*, *28*(6), 1923–1932.

Innes, R. J., Howard, Z. L., Thorpe, A., Eidels, A., & Brown, S. D. (2021). The effects of increased visual information on cognitive workload in a helicopter simulator. *Human factors*, *63*(5), 788–803. doi: https://doi.org/10.1177/0018720820945409

Strayer, D. L., Cooper, J. M., Turrill, J., Coleman, J. R., & Hopman, R. J. (2015). Measuring cognitive distraction in the automobile iii: A comparison of ten 2015 in-vehicle information systems. .

Thorpe, A., Nesbitt, K., & Eidels, A. (2020, oct). A systematic review of empirical measures of workload capacity. *ACM Trans. Appl. Percept.*, *17*(3). doi: https://doi.org/10.1145/3422869

Wickens, C. D. (2002). Multiple resources and performance prediction. *Theoretical issues in ergonomics science*, *3*(2), 159–177. doi: 10.1080/14639220210123806
